# Predictive model for daily risk alerts in sepsis patients in the ICU: visualization and clinical analysis of risk indicators

**DOI:** 10.1093/pcmedi/pbaf003

**Published:** 2025-02-08

**Authors:** Hao Yang, Jiaxi Li, Chi Zhang, Alejandro Pazos Sierra, Bairong Shen

**Affiliations:** Information Center, West China Hospital of Sichuan University, Chengdu 610041, China; Department of Computer Science and Information Technologies, Research Center for Information and Communications Technologies, University of A Coruña, Biomedical Research Institute of a Coruña, A Coruña 15071, Spain; Department of Clinical Laboratory Medicine, Jinniu Maternity and Child Health Hospital of Chengdu, Chengdu 610031, China; Joint Laboratory of Artificial Intelligence for Critical Care Medicine, Department of Critical Care Medicine and Institutes for Systems Genetics, Frontiers Science Center for Disease-related Molecular Network, West China Hospital, Sichuan University, Chengdu 610041, China; Department of Computer Science and Information Technologies, Research Center for Information and Communications Technologies, University of A Coruña, Biomedical Research Institute of a Coruña, A Coruña 15071, Spain; Joint Laboratory of Artificial Intelligence for Critical Care Medicine, Department of Critical Care Medicine and Institutes for Systems Genetics, Frontiers Science Center for Disease-related Molecular Network, West China Hospital, Sichuan University, Chengdu 610041, China

**Keywords:** sepsis, Transformer, time-series, visualization, predicting mortality

## Abstract

This study introduces a novel Transformer-based time-series framework designed to revolutionize risk stratification in Intensive Care Units (ICUs) by predicting patient outcomes with high temporal precision. Leveraging sequential data from the eICU database, our two-stage architecture dynamically captures evolving health trajectories throughout a patient’s ICU stay, enabling real-time identification of high-risk individuals and actionable insights for personalized interventions. The model demonstrated exceptional predictive power, achieving a progressive AUC increase from 0.87 (±0.021) on admission day to 0.92 (±0.009) by day 5, reflecting its capacity to assimilate longitudinal physiological patterns. Rigorous external validation across geographically diverse cohorts—including an 81.8% accuracy on Chinese sepsis data (AUC=0.73) and 76.56% accuracy on MIMIC-IV-3.1 (AUC=0.84)—confirmed robust generalizability. Crucially, SHAP-derived temporal heatmaps unveiled mortality-associated feature dynamics over time, bridging the gap between model predictions and clinically interpretable biomarkers. These findings establish a new paradigm for ICU prognostics, where data-driven temporal modeling synergizes with clinician expertise to optimize triage, reduce diagnostic latency, and ultimately improve survival outcomes in critical care.

## Graphical Abstract

**Graphical Abstract:**
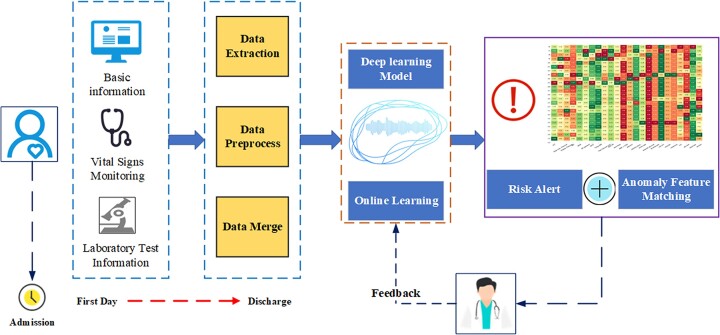
The framework of hospital system integration with predictive models.

## Introduction

Sepsis is defined as life-threatening organ dysfunction resulting from a dysregulated host response to infection, as outlined in the Sepsis-3 definition [1]. It is a critical medical emergency and clinical syndrome characterized by widespread inflammation. Sepsis can also lead to disseminated intravascular coagulation [2], causing multiple organ failures, high mortality rates, and long-term disability among survivors. Sepsis is most commonly observed in critically ill patients, with in-hospital mortality ranging from 20 to 50% [3]. Despite continuous advancements in medical technology that enable timely and effective treatment, a subset of patients still succumbs to the acute progression of sepsis. Therefore, accurately predicting mortality risk in sepsis patients is of paramount clinical importance. Such prognostic predictions can help intensivists make prompt, informed decisions regarding interventions, ultimately improving survival outcomes for sepsis patients.

Accurately predicting mortality among sepsis patients is crucial for clinical physicians, as it facilitates not only the evaluation of disease severity but also the optimization of treatment strategies, reduction in adverse outcomes, and extension of patient longevity. Presently, various clinical scoring systems, such as the sequential organ failure assessment score [4] and the acute physiology and chronic health evaluation (APACHE-II) scoring system [5], assist clinicians in evaluating sepsis severity and predicting adverse events. Nonetheless, these scoring systems are devised for the broader population of critically ill patients and do not specifically target sepsis. Given the critical nature of sepsis, the diagnosis and treatment are of great importance, and an early pre-sepsis diagnosis could potentially enhance patient survival rates.

Recently, machine learning in medical research has facilitated the development of predictive models customized to specific clinical requirements and data characteristics, exhibiting superior predictive performance in anticipating adverse outcomes compared to conventional clinical scoring systems. The construction of existing sepsis mortality prediction models predominantly relies on the utilization of machine learning models, like logistic regression, random forest, and XGBoost [6], and achieved a good area under the receiver operating characteristic curve (AUC) in the range 0.70–0.85 [7–9]. However, there is still considerable potential to improve their performance.

The majority of research has focused on constructing predictive models using data from isolated instances or patient-specific time intervals, inadvertently overlooking the temporal evolution of underlying disease conditions. In this study, we utilized the eICU Collaborative Research Database [10], focusing on patients diagnosed with sepsis, and conducted experiments by extracting relevant data such as vital signs and laboratory test results related to sepsis. Through organizing this data chronologically and analyzing the statistical characteristics and temporal patterns of the time-series samples, we constructed a time-series model based on the Transformer architecture [11] to predict mortality risk in sepsis patients. Furthermore, visual algorithms were used to analyze activation characteristics at different time intervals to explore their correlation with the risk of sepsis-related death. Our objective is to provide novel insights and approaches for the treatment and prognosis of sepsis by establishing a temporal model.

## Methods and materials

### Participants

The eICU Collaborative Research Database is a comprehensive, multi-center intensive care unit (ICU) dataset developed through a collaboration between the Massachusetts Institute of Technology and the Philips Group. It contains high-quality clinical data from >200 000 patients admitted to 208 hospitals across the USA, spanning the years 2014 to 2015. The dataset includes diverse information such as demographics, vital signs, laboratory results, treatments, diagnoses, and more, with its reliability validated through multiple research studies [10].

The study included patients diagnosed with sepsis, as recorded in the diagnostic table of the eICU database. Exclusion criteria were as follows: patients under 18 years of age, those with unknown gender, cases where the diagnosis was deemed invalid at discharge, patients with ICU stays of <24 h, and records with >30% missing data.

**Figure 1.**
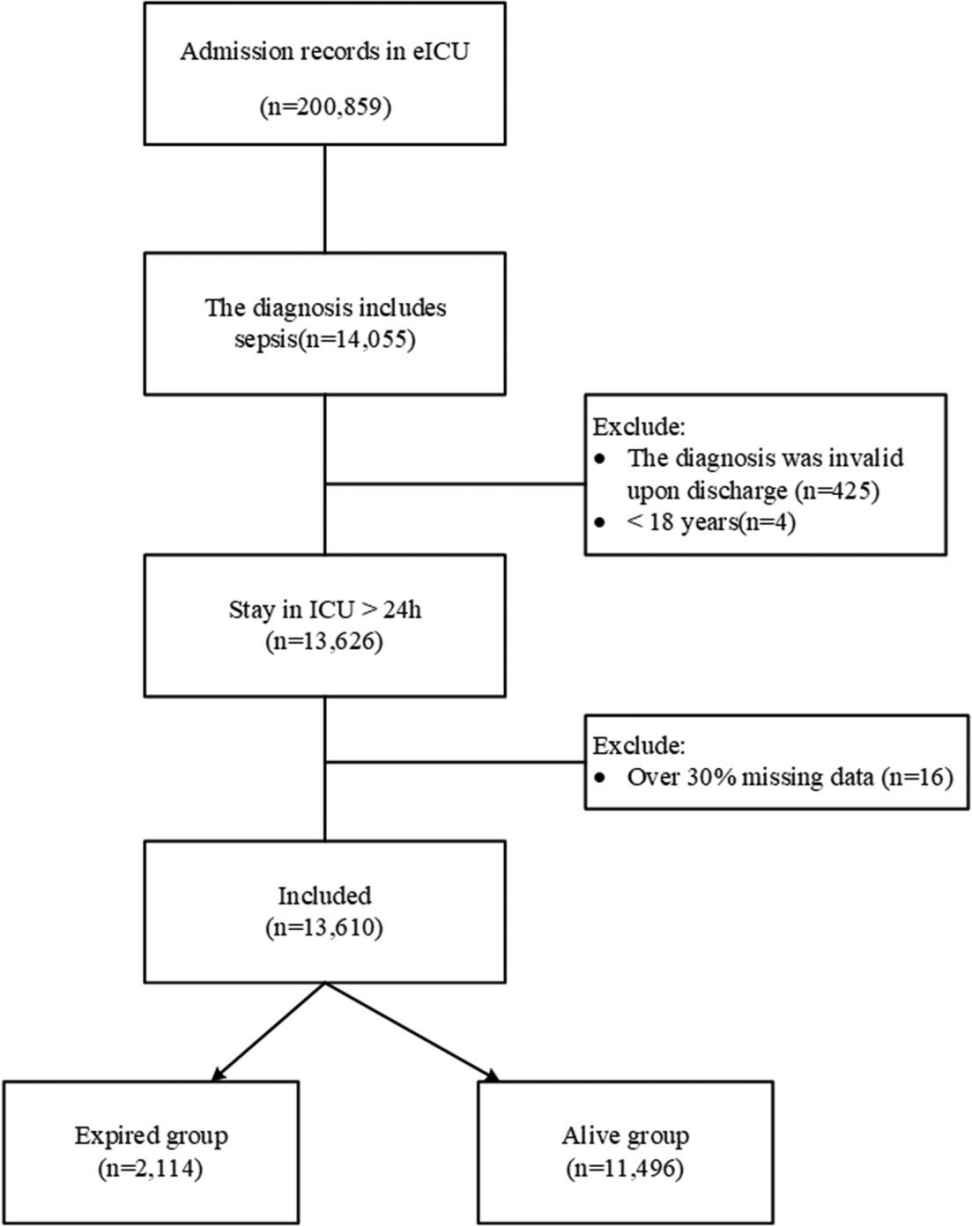
Flowchart of data inclusion and exclusion criteria for ICU sepsis patients in the eICU database.

In this study, mortality was defined as in-hospital mortality, referring to deaths that occurred during the hospital stay as documented in the discharge disposition. The participants' screening workflow is detailed in Fig. 1.

### Data collection

Drawing upon clinical expertise, published literature, and data available in the eICU database, we gathered the following categories of data: (i) demographic information encompassing gender, age, and ethnicity; (ii) vital signs recorded post-ICU admission, including heart rate, mean arterial pressure, respiratory rate, and oxygen saturation; and (iii) laboratory test results obtained after ICU admission, such as creatinine and hemoglobin levels, among others. In total, there are 226 distinct vital sign data features (see supplementary Table 1 in the online supplementary material for further details).

### Data preprocessing

Using Python data preprocessing libraries such as NumPy and pandas [12,13], the data are organized chronologically based on the ICU admission timeline, utilizing the ‘offset’ field in each eICU data table. With 24 hourly sampling points per day, a total of 226 features are considered, and for each hourly sampling point, the nearest available data value is selected. This results in a time-series matrix of size 24 × 226. To prioritize early-stage risk prediction, the model focuses on the first 5 days of ICU admission, as sepsis-related mortality is most pronounced within this period. This design is informed by clinical observations that the majority of sepsis-related mortalities occur early in the ICU stay, making early identification of high-risk patients crucial for improving outcomes. For patients with stays >5 days, a sliding time-window approach ensures that the model remains dynamic, continuously updated with patient data throughout the ICU stay. The data is randomly divided into training, validation, and testing sets in a 7 : 2 : 1 ratio to ensure robust model performance.

### Missing data and filling

In time-series analysis, missing data is a common challenge. Given the presence of missing data in our study and the limitations of traditional imputation methods, we employed two distinct imputation approaches tailored to the nature of the data. For the temporal data, particularly the daily risk indicators of sepsis patients in the ICU, we utilized forward imputation. This method is well-suited for time-series data, as it assumes that the missing values are likely to be similar to the preceding values, preserving the temporal continuity of the data. For other basic features that are not time-dependent, we opted for random forest imputation. This method is particularly effective for multivariate datasets, as it captures complex, non-linear relationships among features to predict missing values. By leveraging interactions between variables, random forest imputation provides accurate estimates, making it ideal for filling in missing data in non-sequential features.

These imputation strategies were chosen to address the specific needs of our time-series analysis and ensure that missing data would not bias our model. The combination of forward imputation for temporal features and random forest imputation for other attributes helped to improve the overall performance of the predictive model.

### Analysis platform

We utilized the deep learning framework PyTorch (https://pytorch.org/) to construct our prediction model. PyTorch offers a comprehensive range of functionalities and empowers users with complete control over their Python programs [14]. Our prediction model is built on a Windows computer with the following specifications: Windows 11 operating system, 12th Gen Intel (R) Core (TM) i7-12700F central processing unit, 32 GB random access memory, and NVDIA GeForce RTX3060Ti graphics processing unit.

### Model architecture

The proposed model is a two-stage Transformer-based architecture designed to capture both hourly and daily temporal patterns in patient time-series data. The input data consists of 226 vital sign features recorded every hour across a maximum of 5 days, with variable time lengths depending on the patient’s ICU stay. In the first stage, the model processes the hourly time-series data for each day using an hour-level Transformer encoder. For each day, the input data, which has a shape of 'batch size, days, features, hours' is reshaped into hourly sequences of dimension 'batch size × days, hours, features'. The Transformer encoder, comprising multiple self-attention layers, is applied to these hourly sequences to capture dependencies across hours within a day. The output of the hour-level Transformer represents the encoded hourly features for each day. These encoded features are then aggregated using average pooling across hours to produce a daily representation.

The second stage of the model processes the daily representations generated by the hour-level Transformer using a day-level Transformer encoder, which is designed to model temporal dependencies across up to 5 days. Regardless of the actual number of ICU days per patient, the input for this module is standardized to 5 days. For patients with <5 days of data, masking is applied to the missing days. This ensures that the model can handle patients with different ICU stay durations while maintaining temporal consistency across the entire cohort.

The use of masking in this context is essential. Missing data for these patients may result from several possible scenarios: data may be absent due to technical issues, incomplete records, or other factors; the patient may have passed away before reaching the fifth day in the ICU; the patient may have been discharged early following recovery; or the patient may have been transferred to another hospital or department. In all such cases, masking ensures that these missing days do not introduce bias or distort the analysis. By masking, the model avoids making assumptions about the reason for the missing data—whether it is due to data absence or patient mortality—thereby preserving the integrity of the model and avoiding skewed results. Masking also maintains the temporal structure of the data, as all patients are processed within the same standardized 5-day window, regardless of their actual ICU stay. This allows the model to effectively capture both intra-day and inter-day dependencies without discarding valuable information from patients with shorter stays. Moreover, by masking the missing data, the model avoids learning from potentially misleading information, especially when the absence of data could be correlated with critical patient outcomes such as mortality.

The final output from the day-level Transformer, corresponding to the 5th day, is used as the patient's overall time-series representation. This representation is then passed through a fully connected layer to produce the final prediction, which is a continuous value representing the predicted outcome. By fixing the day-level Transformer to 5 days, the model remains flexible enough to handle patients with varying ICU stay durations while ensuring temporal consistency across different patient trajectories.

**Figure 2.**
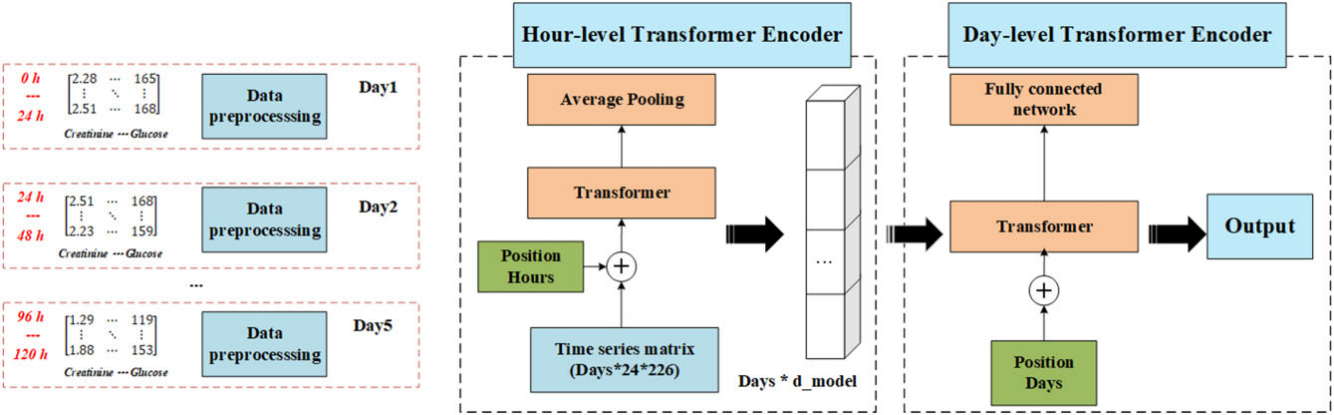
Framework of the two-stage Transformer model for ICU patient time-series analysis: hourly and daily temporal pattern encoding with prediction.

This two-stage Transformer architecture, which effectively captures both intra-day and inter-day temporal patterns over a maximum of 5 days, is well-suited for modeling the complex trajectories of patients in critical care settings. The overall framework is illustrated in Fig. 2. (Please refer to the following link for the relevant code: https://github.com/yanghaoljx/Sepsis-ICU-timeseries)

Based on the classification results of the model, we performed a visual analysis using SHAP [15] and obtained the corresponding heatmap of feature weights. Considering the problem of imbalanced positive and negative samples in the study, the focal loss [16] is introduced as the loss function for the model.

## Results

### Baseline

A total of 13  610 patients were selected from the eICU database for inclusion in the study. Of these, 2 114 patients died during their ICU stay, while 11  496 patients survived. Data extraction was performed using the PostgreSQL database system [17]. Statistical analysis was carried out using SPSS 22.0 [18], while data cleaning, model construction, and performance evaluation were performed using Python. Continuous variables are presented as the median (interquartile range) and categorical data are shown as counts (percentages). The Mann–Whitney U test [19] was used to analyze continuous variables and the chi-square test [20] was applied to assess significant differences in categorical variables. The results are presented in Table 1.

**Table 1.** Baseline characteristics and statistical comparisons of ICU sepsis patients; continuous variables are expressed as mean (standard deviation) and categorical variables are expressed as counts (percentages).

Characteristic		Alive	Expired	*P-*valu*e*
ICU stay [mean (SD)]		4.171 (5.340)	5.518 (7.933)	<0.001^a^
Apache II [mean (SD)]		66.020 (23.845)	88.479 (30.147)	<0.001^a^
Gender (%)	Female	5693 (49.52)	1004 (47.49)	0.1889^b^
	Male	5801 (50.46)	1110 (52.51)	
	Unknown	2 (0.02)	0 (0.00)	
Age, years [mean (SD)]		65.252 (16.309)	69.807 (13.987)	<0.001^a^
Ethnicity (%)	African–American	1136 (9.88)	222 (10.50)	0.4997^b^
	Asian	175 (1.52)	43 (2.03)	
	Caucasian	9080 (78.98)	1641 (77.63)	
	Hispanic	425 (3.70)	88 (4.16)	
	Native American	94 (0.82)	17 (0.80)	
	Other/unknown	586 (5.10)	103 (4.87)	
Height [mean (SD), cm]		168.341 (14.618)	168.067 (14.465)	0.428^a^
Unit type (%)	Cardiac ICU	741 (6.45)	155 (7.33)	0.0143^b^
	CCU-CTICU	697 (6.06)	121 (5.72)	
	CSICU	132 (1.15)	33 (1.56)	
	CTICU	85 (0.74)	18 (0.85)	
	Med-Surg ICU	7669 (66.71)	1345 (63.62)	
	MICU	1510 (13.14)	331 (15.66)	
	Neuro ICU	222 (1.93)	41 (1.94)	
	SICU	440 (3.83)	70 (3.31)	
Weight [mean (SD), kg]		83.271 (27.945)	79.428 (27.426)	<0.001^a^

CCU-CTICU (Coronary Care Unit-Cardiothoracic ICU) CSICU (Cardiothoracic Surgical ICU) CTICU (Cardiothoracic ICU) Med-Surg ICU (Medical-Surgical ICU) MICU (Medical ICU) SICU (Surgical ICU)

a Continuous variables were compared using the Mann–Whitney U test.

b Categorical variables were compared using the chi-square test.

### Model performance

**Figure 3.**
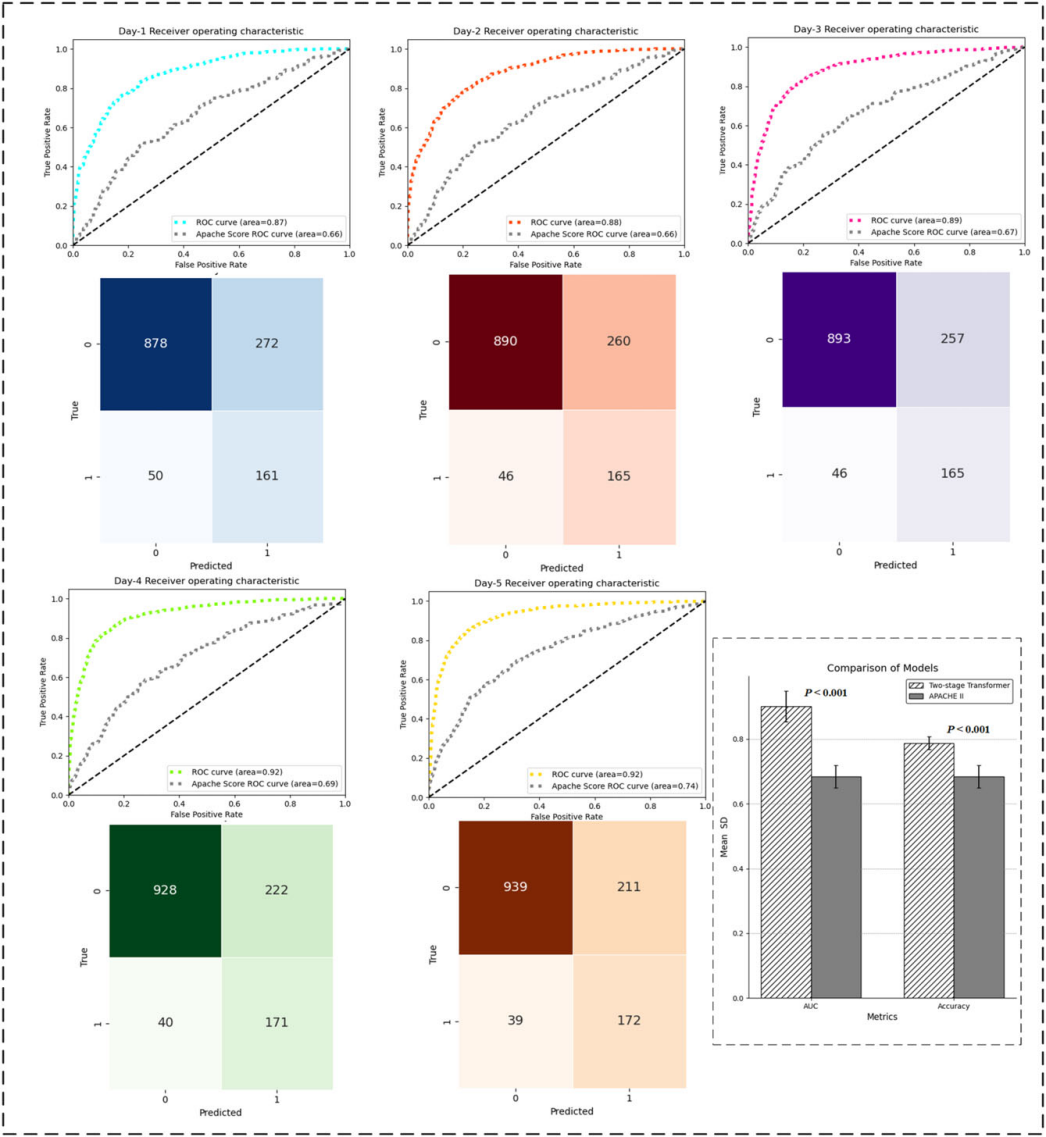
Receiver operation characteristic (ROC) curves, confusion matrix, and statistical comparison (*P* < 0.001) demonstrating the superior predictive performance of the two-stage Transformer model over APACHE II across ICU days. Statistical comparison was performed using the Mann–Whitney U Test.

Based on the temporal features observed for patients each day, a two-stage Transformer network model was developed to enhance prediction accuracy for sepsis outcomes. This model integrates daily time-series data, effectively capturing temporal dependencies and improving overall predictive performance. It is observed that the model performs optimally in predicting outcomes for the fifth day. For each day's prediction, the model utilizes continuous data from the preceding days to estimate the risk of in-hospital mortality. For example, the Day 3 model uses a masked matrix, applying masking to the subsequent day's data, and relies on data from Day 1 and Day 2 to predict the risk of in-hospital mortality. The model then directly outputs the predicted mortality risk for the patient. A comparison, presented in Table 2 and Fig. 3, illustrates the daily prediction results, alongside those obtained using the traditional APACHE II-based prediction method. Additionally, clearer daily activation heatmaps are provided in supplementary Figs. 1–5, see online supplementary material. The results, derived from the testing data, include 211 samples from the expired group and 1 150 samples from the alive group.

**Table 2.** Performance comparison [mean (SD)] of the two-stage Transformer model and APACHE II in predicting in-hospital mortality across ICU days.

Model	Day 1	Day 2	Day 3	Day 4	Day 5	APACHE II
AUC	0.87 (0.021)	0.88 (0.022)	0.89 (0.019)	0.92 (0.011)	0.92 (0.009)	0.68 (0.030)
Accuracy (%)	77.82 (1.63)	78.93 (1.53)	80.98 (0.89)	82.03 (0.71)	83.92 (0.69)	63.74 (1.78)
F1-score^a^	0.704 (0.058)	0.711 (0.061)	0.732 (0.044)	0.773 (0.048)	0.804 (0.024)	0.667 (0.045)

a F1-score, harmonic mean of precision and recall.

A comparison with existing models, such as decision tree [21], XGBoost, and Long Short-Term Memory (LSTM) [22], reveals that the two-stage Transformer network significantly outperforms in predicting sepsis prognosis. To facilitate the input for traditional machine learning models, daily feature values are averaged over the 24-h period, resulting in a consistent 226-dimensional feature vector per day. This approach ensures uniform input dimensions across all data points while also reducing computational complexity. The mean and variance of the model's performance across different days were calculated (a bar chart comparing the results of different models is presented in supplementary Fig. 6, see online supplementary material), with the specific results presented in Table 3.

**Table 3.** Comparative performance metrics [mean (SD)] of predictive models for sepsis prognosis: decision tree, XGBoost, Multilayer Perceptron (MLP), LSTM, and two-stage Transformer.

Metric	Decision tree	XGBoost	MLP	LSTM	Two-stage Transformer	*P*-value
AUC	0.733 (0.035)	0.727 (0.045)	0.905 (0.015)	0.876 (0.025)	0.92 (0.009)	<0.001
Accuracy (%)	72.35 (1.68)	66.46 (2.66)	81.35 (0.89)	78.03 (1.23)	83.92 (0.69)	<0.001
F1-score^a^	0.750 (0.045)	0.645 (0.063)	0.801 (0.035)	0.790 (0.039)	0.804 (0.024)	<0.001

a F1-score, harmonic mean of precision and recall.

**Figure 4.**
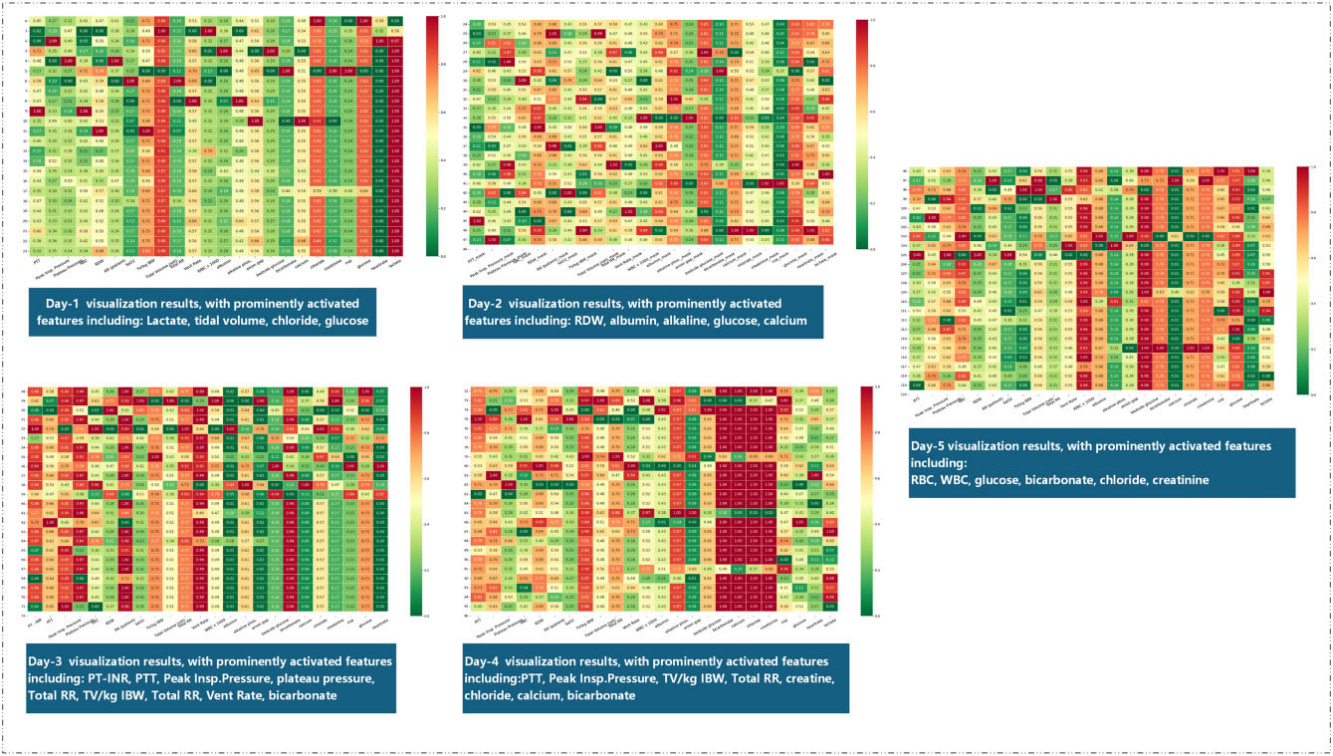
Daily feature visualization heatmaps showing feature activation in the two-stage Transformer model. The vertical axis represents the 24 h of the day, with the color intensity indicating the level of activation, where redder colors correspond to higher activation levels. (Day 5: key activated features include RBC, WBC, glucose, bicarbonate, chloride, and creatinine, which are crucial for assessing overall blood and metabolic status; Day 4: PTT, peak insp. pressure, TV/kg IBW, total RR, creatine, chloride, calcium, and bicarbonate, indicating vital respiratory and metabolic indicators; Day 3: PT-INR, PTT, peak insp. pressure, plateau pressure, total RR, TV/kg IBW, vent rate, and bicarbonate, highlighting coagulation, ventilation, and acid–base balance; Day 2: RDW, albumin, alkaline, glucose, and calcium, reflecting red blood cell characteristics and nutritional status; Day 1: lactate, tidal volume, chloride, and glucose, important for early sepsis detection and metabolic evaluation). RBC, Red blood cells; WBC, white blood cells; glucose, blood sugar level; PTT, partial thromboplastin time; peak insp. pressure, peak inspiratory pressure; TV/kg IBW, tidal volume per kilogram ideal body weight; total RR, total respiratory rate; PT-INR, prothrombin time—international normalized ratio; RDW, red cell distribution width.

Utilizing the prediction model, weight analysis is conducted using the SHAP algorithm, which generates visual heatmaps. Due to limitations in formatting, the paper presents only the heatmap representing the most prominently activated feature for each day, as depicted in Fig. 4).

### External validation

To assess the generalizability of the model, we utilized ICU sepsis patient data from a large tertiary hospital collected over the past 3 years (2021–2023). Inclusion criteria required patients to have an ICU stay of ≥5 days and discharge outcomes (survival or mortality) were carefully verified. A total of 417 patients were included, with 350 classified as alive and 67 as expired. The model achieved an overall accuracy of ∼81.8%, with an AUC value of 0.73. To account for potential performance variations due to ethnic differences, we further validated the model using the MIMIC-iv-3.1database [23]. Sepsis patient data were extracted based on ICD-9 codes (‘99591’, ‘99592’, ‘78552’) [24], and outcomes were determined using the ‘date of death (dod)’ column in the patient's table. To ensure the scientific rigor of the validation and the stability of the model's performance, we performed feature selection and randomized grouping of the data. Compared to the domestic dataset, the MIMIC dataset exhibited a more balanced ratio of positive and negative samples, with 877 classified as alive and 1 192 as expired. The model achieved an overall accuracy of 76.56% on this dataset, with an AUC value of 0.84, demonstrating strong predictive performance. The results indicate that while the accuracy was slightly higher on the domestic dataset, the AUC value for the MIMIC dataset was superior, likely due to the larger sample size and the balanced distribution of outcomes. This validation highlights the model's adaptability across diverse populations and data sources, providing a solid foundation for future clinical applications across regions and ethnic groups. Specific results are presented in supplementary Figs. 7 and 8, see online supplementary material.

### Clinical application

In our research, we are investigating the integration of real-time patient data from hospital information systems to enhance the assistance provided to clinical sepsis patients. Our system integration framework involves collecting essential patient information, laboratory test results, and vital signs from the point of admission and consolidating this data in a centralized data center. Given that patients generate time-series data every minute, continuous sepsis risk assessment is impractical. Therefore, we process this data through an application programming interface to utilize deep learning models, which generate daily risk predictions until the patient is discharged. To improve the model's adaptability, we plan to incorporate online learning techniques, allowing the model to adjust in real-time based on clinical feedback. These predictions are then relayed back to the patient monitoring system (shown in supplementary Fig. 9, see online supplementary material). Ultimately, the decision to intervene clinically is made by the doctor, thus completing the closed-loop process. Currently, we have only completed the framework design, and further development is needed for data and system integration to facilitate clinical application.

## Discussion

Sepsis represents a critical and life-threatening condition that requires careful identification of patients at high risk in order to optimize treatment and prognostic predictions. In this study, we propose a two-stage Transformer model, which demonstrates significant advantages in predicting sepsis outcomes for ICU patients. As the length of ICU stay increases, the model's predictive performance gradually improves. Notably, the AUC reaches 0.92 (±0.009) on Day 5, significantly higher than the AUC of 0.87 (±0.021) on Day 1. This trend indicates that the model better captures the time-dependent nature of patients' condition changes over time, and as more clinical data accumulates, the reliability of its predictions increases. From an architectural perspective, the Transformer network incorporates both hourly and daily time-series patterns. This allows it to effectively capture dependencies across both hours and days, which is crucial given the frequent fluctuations in ICU patients' vital signs. The two-stage Transformer not only tracks within-day trends in vital signs but also models cross-day time-series data, identifying signs of deterioration that emerge gradually during the hospital stay.

In comparison with traditional models such as the APACHE II scoring system and other models (Tables 2 and 3), our model consistently outperforms across various metrics. For instance, on Day 5, the AUC reached 0.92 (±0.009), significantly surpassing traditional machine learning models like XGBoost (AUC of 0.7277) and decision trees (AUC of 0.7329). Similarly, in terms of accuracy and F1 score, the Transformer model exhibited more stable and superior performance. Particularly, the F1 score on day 5 was 0.804 (±0.024), outperforming deep learning models such as MLP (0.801) and LSTM (0.790). These results suggest that the Transformer architecture not only excels in accurately predicting sepsis outcomes but also has a strong ability to adapt to the dynamic changes in patient conditions, making it well-suited for real ICU environments. Furthermore, by employing the SHAP visualization algorithm, we noted a rising presence of life features strongly linked to mortality as the time sequence advances. This is distinctly evident from their elevated levels of activation. Comparing these obtained activation features with relevant literature, we found that these patterns align with known clinical markers of sepsis progression, demonstrating their potential clinical and research value.

The vital signs and diagnostic indicators recorded during the patient's initial day of admission must not be overlooked as they play a pivotal role in evaluating the severity of the patient's condition. According to our model, on the first day after ICU admission, lactate levels were the most strongly associated feature with patient mortality, followed by tidal volume, chloride, bedside glucose, and ideal body weight (IBW). In most cases, elevated lactate levels were correlated with poor prognosis, particularly in sepsis, trauma, bleeding, shock, and cardiac arrest [25–27]. Lactate level possesses diverse diagnostic applications and has been extensively utilized as an indicator for resuscitation, the stratification of risk, and the prediction of mortality in cases of sepsis [28]. In a prospective cohort study of patients with infections in the emergency department, elevated lactate levels were associated with increased mortality, and an initial lactate level ≥4 mmol/l was found to be linked to a 28% in-hospital mortality rate [29]. ICU patients who require mechanical ventilation face a high incidence and mortality rate [30, 31]. Acute respiratory distress syndrome (ARDS) contributes to the elevated admission and mortality rates in the ICU, and lung-protective ventilation, including low tidal volume ventilation, has demonstrated benefits for both ARDS and non-ARDS patients [32, 33]. According to our model, in addition to lactate concentration, electrolyte disturbances are also common in the ICU. Serum chloride, the second most abundant electrolyte in the human body, plays a critical role in the pathophysiology of acute heart failure and is considered a therapeutic target for reducing mortality [34–36]. Among ICU patients with heart failure, various electrolyte abnormalities were commonly observed, with hyponatremia and hypochloremia being the most prevalent [37]. Previous studies have investigated the detrimental effects of hyperglycemia on the prognosis of critically ill patients, such as those with sepsis, myocardial infarction, acute pancreatitis, and stroke [38]. Our model enables us not only to observe the indicators connected with human organ failure, but also systemic indicators like glucose levels. In our research, serum blood glucose concentration during ICU stay was associated with overall ICU mortality in sepsis patients. Patients with severe hyperglycemia (≥200 mg/dl) upon ICU admission exhibit the highest overall ICU mortality, regardless of whether they have diabetes, highlighting sustained hyperglycemia as a significant risk factor for ICU mortality in sepsis patients [39].

As the disease advances and in response to the subsequent administration of relevant medications, surgical interventions, and other treatment modalities, the patient's indicators will undergo alterations. With the model, on the second day the levels of red cell distribution width (RDW), albumin, alkaline phosphatase, and calcium start to increase. Following respiratory support and basic care, most patients show signs of recovery. However, patients with organ failure begin to exhibit disruptions in relevant organ function indicators. RDW is a parameter traditionally used for the differential diagnosis of anemia, and its increase reflects disruptions in red blood cell production and unregulated red cell homeostasis. Tutak and Avni Findiki conducted a study indicating that elevated RDW levels serve as a reliable marker for predicting mortality and should be included in the APACHE II score to forecast patient outcomes [40]. RDW values above the upper limit of 14.5% are associated with abnormal metabolic conditions such as inflammation, oxidative stress, and nutritional imbalances [41]. Özdemir *et al*. demonstrated an association between high RDW levels and mortality in ICU patients with mixed conditions [42]. Similarly, Safdar *et al*. reported a correlation between high RDW levels and 30-day mortality rates in follow-up patients from ICU medical and surgical departments [43]. Salmoran *et al*. also found a correlation between elevated RDW levels and mortality in ICU patients [44]. From this, the RDW in routine blood tests plays a significant role in predicting the mortality rate and prognosis of ICU sepsis patients and should not be overlooked. Moreover, our model showed another indicator associated with sepsis severity, i.e. albumin, a multifunctional protein with colloidal and pharmacological properties that exhibits physiological functions intricately linked to its distribution (intravascular, extravascular, and intracellular locations), concentration, and complex structure [45]. Yin *et al*. conducted a prospective cohort study and found that serum albumin levels <2.92 g/dl upon admission are associated with an increased 28-day mortality rate in patients with severe sepsis [46]. Research suggested that serum albumin trends can predict mortality rates in ICU sepsis patients. Significant correlations exist between mortality rates and serum albumin trends, as well as average, peak, admission, and minimum levels of albumin [47]. In critically ill elderly patients, serum albumin levels upon ICU admission serve as effective predictive indicators for mortality and other outcomes. Low serum albumin levels upon admission were independent risk factors for 6-month mortality in critically ill elderly patients after ICU discharge [48]. Sepsis is associated with organ damage. Acute kidney injury (AKI) is one of the most common organ failures. Total alkaline phosphatase activity slightly increases in ICU patients with septic AKI, and its activity positively correlates with ICU length of stay [49]. In a predictive experiment for early liver failure in pediatric ICU patients, elevated alkaline phosphatase on the first day of admission was identified as a key laboratory parameter for diagnosing liver dysfunction. Alkaline phosphatase is widely used to predict liver function prognosis in ICU patients [50].

The parameters most strongly associated with ICU mortality were identified, with one-third of sepsis-related deaths occurring within 3 days of ICU admission [51]. In the models on the third and fourth days, coagulation parameters such as prothrombin time (PT) and activated partial thromboplastin time (APTT) reflect their value in predicting the length of hospital stay for septic patients. PT and APTT can serve as early prognostic markers for severe pneumonia requiring transfer to the ICU. Baranovskii et al. compared these coagulation parameters and various coagulation and inflammation factor levels between patients with early treatment-resistant respiratory failure or severe acute respiratory distress syndrome requiring ICU treatment and stable COVID-19 patients. They discovered that early measurement of PT values can predict the course of COVID-19-related pneumonia. Prolonged PT in patients transferred to the ICU and PT levels upon admission for COVID-19 patients can serve as early prognostic markers for severe pneumonia requiring ICU care [52, 53]. APTT, the most used and sensitive screening method for assessing clotting activity, has been shown in multiple studies to be a risk factor for thrombosis and pulmonary embolism. It is a significant predictor for pulmonary embolism in ICU patients [54–57]. Monitoring APTT in ICU patients should be emphasized to prevent pulmonary embolism. For patients admitted to the ICU due to respiratory conditions, particularly COVID-19, respiratory-related parameters exhibit a strong association with ICU mortality. Plateau pressure, which represents airway pressure within the alveoli during positive pressure ventilation when the breath is held, has been analyzed for its impact on the prognosis of patients with ARDS. There is a correlation between plateau pressure and short-term mortality, with significantly higher overall short-term mortality rates observed in patients with plateau pressure >32 cm H_2_O within 3 days of ICU admission. This correlation becomes evident in the following days and may persist for the first 3 days after ICU admission [58]. The respiratory index, the ratio of peripheral oxygen saturation (SpO_2_) to respiratory rate (RR), has been validated for prognostic stratification in patients with acute pulmonary embolism [59]. However, in patients admitted to the ICU due to COVID-19, RR may be an important parameter that has received insufficient reporting and attention. Marziti and Becattini found that RR is a better predictor of mortality compared to blood gas parameters PaO₂/FiO₂ ratio (P/F) and SpO₂/FiO₂ ratio (STP/F)in evaluating patients with COVID-19-related pneumonia admitted to the ICU [60].

In the model on the fifth day, compared to the previous four days, red blood cell, white blood cell, and creatinine levels began to rise. Cells of the innate immune system and adaptive immune system play a crucial role in the host response to sepsis, which is associated with profound inhibition of constitutive neutrophil apoptosis [61]. During the onset of sepsis, neutrophils respond rapidly to infection, their numbers increase sharply, and they quickly migrate to sites of severe infection [62]. While neutrophil counts are indicative of the overall inflammation severity, in intricate sepsis scenarios, the delay in neutrophil apoptosis could result in a persistent elevation of neutrophil counts [61]. Hence, the significance of leukocytes as an indicator should not be diminished in the presence of sepsis; rather, it should continue to be closely monitored. The incidence of AKI in sepsis patients in the ICU can reach up to 60%, and the occurrence of AKI is associated with increased mortality, with survivors being at risk of developing chronic kidney disease [63, 64]. Serum creatinine slowly increases after the onset of AKI, and its rise can be further delayed due to large fluid resuscitation and fluid balance [65]. On the third day following sepsis-induced AKI, serum creatinine may only increase to the borderline value. If the glomerular filtration rate remains stable, the expansion of distribution volume is expected to lead to a decrease in serum creatinine levels, resulting in a delay in the number of days before serum creatinine concentration rises [66]. Therefore, continuous monitoring of creatinine levels in sepsis patients in the ICU allows for better assessment of renal injury.

We chose Day 5 as the key time point for prediction because the highest mortality risk in sepsis patients typically occurs within around the fifth day of ICU admission [67, 68]. Studies have consistently shown that early identification of high-risk patients during this window significantly enhances the chances of better clinical outcomes [69, 70]. For patients staying beyond 5 days in the ICU, the model can still be applied by utilizing a sliding time-window approach. This method allows the model to use the most recent data within a defined window, thereby maintaining predictive performance.

Although this study demonstrates the significant advantages of Transformer-based models in predicting the prognosis of sepsis patients in the ICU, it also has some limitations. A primary challenge is the sparsity of the data, which stems from the inherent characteristics of time-series data. Variability in patient treatment regimens and the frequency of diagnostic tests during the ICU stay contribute to this issue. As discrepancies in treatment protocols and testing schedules accumulate over time, data gaps become increasingly evident. To address this, we employed a sliding-window approach combined with filling missing values using the most recent available data points, aiming to generate more complete time-series matrices. This strategy helps mitigate data gaps while minimizing potential distortion. However, it may still introduce some degree of data distortion, particularly when there are substantial differences in treatment protocols or testing frequencies. Additionally, the data used in this study was derived from a single center, which may limit the generalizability of the model, particularly in different regions, populations, or disease conditions. Therefore, the model's performance may vary across hospitals and diverse patient demographics, including differences in race, age, and gender. While the model has shown high predictive accuracy, its performance remains constrained by the quality and completeness of the input data, particularly its sensitivity to missing data and noise. We are currently collecting real-world data to validate and further optimize the model, with the aim of improving its generalizability and robustness across diverse clinical settings. However, this process will take time and may still encounter challenges related to data integration and quality control.

## Conclusion

The results of this study highlight the effectiveness of time-series algorithms, specifically the Transformer-based approach, in predicting patient outcomes in the ICU. The utilization of these algorithms enables the capture of temporal patterns and the integration of comprehensive patient features, leading to improved accuracy and reliability in predictions. Furthermore, the identification and activation of life features associated with mortality provide valuable insights into the underlying mechanisms of sepsis and its progression.

In future research, it would be beneficial to explore the clinical implications of these identified activation features and examine their potential role in guiding early interventions and personalized treatment plans for sepsis patients. Additionally, advancing the development of more sophisticated time-series models by incorporating additional data sources like MetaSepsisBase [71] and refining feature selection techniques holds promise for further enhancing the predictive capabilities and practical applications of mortality prediction in critical care settings.

## Supplementary Material

pbaf003_Supplemental_File

## Data Availability

The data used in this study were sourced from the publicly available eICU database (https://eicu-crd.mit.edu/about/eicu/). For access to the related code, please contact the corresponding author.
