# Supplementary material for: Predictive model for daily risk alerts in sepsis patients in the ICU: visualization and clinical analysis of risk indicators
Source: Precis Clin Med. 2025 Feb 8;8(1):pbaf003. doi: 10.1093/pcmedi/pbaf003 (PMC11878768; doi:10.1093/pcmedi/pbaf003)
Supplement: pbaf003_Supplemental_File [file pbaf003_supplemental_file.docx]

# Supplementary

**Table 1** Features Table for eICU data

| No | Feature | No | Feature | No | Feature |
| --- | --- | --- | --- | --- | --- |
| 0 | -basos | 1 | -eos | 2 | -lymphs |
| 3 | -monos | 4 | -polys | 5 | ALT (SGPT) |
| 6 | AST (SGOT) | 7 | BUN | 8 | Base Excess |
| 9 | Exhaled MV | 10 | FiO2 | 11 | HCO3 |
| 12 | Hct | 13 | Hgb | 14 | LPM O2 |
| 15 | MCH | 16 | MCHC | 17 | MCV |
| 18 | MPV | 19 | Mean Airway Pressure | 20 | O2 Sat (%) |
| 21 | PEEP | 22 | PT | 23 | PT - INR |
| 24 | PTT | 25 | Peak Insp. Pressure | 26 | Plateau Pressure |
| 27 | RBC | 28 | RDW | 29 | RR (patient) |
| 30 | SaO2 | 31 | TV/kg IBW | 32 | Tidal Volume (set) |
| 33 | Total RR | 34 | Vent Rate | 35 | WBC x 1000 |
| 36 | albumin | 37 | alkaline phos. | 38 | anion gap |
| 39 | bedside glucose | 40 | bicarbonate | 41 | calcium |
| 42 | chloride | 43 | creatinine | 44 | cvp |
| 45 | glucose | 46 | heartrate | 47 | lactate |
| 48 | magnesium | 49 | noninvasivediastolic | 50 | noninvasivemean |
| 51 | noninvasivesystolic | 52 | pH | 53 | paCO2 |
| 54 | paO2 | 55 | phosphate | 56 | platelets x 1000 |
| 57 | potassium | 58 | respiration | 59 | sao2 |
| 60 | sodium | 61 | st1 | 62 | st2 |
| 63 | st3 | 64 | systemicdiastolic | 65 | systemicmean |
| 66 | systemicsystolic | 67 | temperature | 68 | total bilirubin |
| 69 | total protein | 70 | troponin - I | 71 | urinary specific gravity |
| 72 | -basos_mask | 73 | -eos_mask | 74 | -lymphs_mask |
| 75 | -monos_mask | 76 | -polys_mask | 77 | ALT (SGPT)_mask |
| 78 | AST (SGOT)_mask | 79 | BUN_mask | 80 | Base Excess_mask |
| 81 | Exhaled MV_mask | 82 | FiO2_mask | 83 | HCO3_mask |
| 84 | Hct_mask | 85 | Hgb_mask | 86 | LPM O2_mask |
| 87 | MCH_mask | 88 | MCHC_mask | 89 | MCV_mask |
| 90 | MPV_mask | 91 | Mean Airway Pressure_mask | 92 | O2 Sat (%)_mask |
| 93 | PEEP_mask | 94 | PT_mask | 95 | PT - INR_mask |
| 96 | PTT_mask | 97 | Peak Insp. Pressure_mask | 98 | Plateau Pressure_mask |
| 99 | RBC_mask | 100 | RDW_mask | 101 | RR (patient)_mask |
| 102 | SaO2_mask | 103 | TV/kg IBW_mask | 104 | Tidal Volume (set)_mask |
| 105 | Total RR_mask | 106 | Vent Rate_mask | 107 | WBC x 1000_mask |
| 108 | albumin_mask | 109 | alkaline phos._mask | 110 | anion gap_mask |
| 111 | bedside glucose_mask | 112 | bicarbonate_mask | 113 | calcium_mask |
| 114 | chloride_mask | 115 | creatinine_mask | 116 | cvp_mask |
| 117 | glucose_mask | 118 | heartrate_mask | 119 | lactate_mask |
| 120 | magnesium_mask | 121 | noninvasivediastolic_mask | 122 | noninvasivemean_mask |
| 123 | noninvasivesystolic_mask | 124 | pH_mask | 125 | paCO2_mask |
| 126 | paO2_mask | 127 | phosphate_mask | 128 | platelets x 1000_mask |
| 129 | potassium_mask | 130 | respiration_mask | 131 | sao2_mask |
| 132 | sodium_mask | 133 | st1_mask | 134 | st2_mask |
| 135 | st3_mask | 136 | systemicdiastolic_mask | 137 | systemicmean_mask |
| 138 | systemicsystolic_mask | 139 | temperature_mask | 140 | total bilirubin_mask |
| 141 | total protein_mask | 142 | troponin - I_mask | 143 | urinary specific gravity_mask |
| 144 | hour_x | 145 | gender | 146 | age |
| 147 | admissionheight | 148 | admissionweight | 149 | hour_y |
| 150 | intubated | 151 | vent | 152 | dialysis |
| 153 | eyes | 154 | motor | 155 | verbal |
| 156 | meds | 157 | ethnicity_African American | 158 | ethnicity_Asian |
| 159 | ethnicity_Caucasian | 160 | ethnicity_Hispanic | 161 | ethnicity_Native American |
| 162 | ethnicity_Other/Unknown | 163 | unittype_CCU-CTICU | 164 | unittype_CSICU |
| 165 | unittype_CTICU | 166 | unittype_Cardiac ICU | 167 | unittype_MICU |
| 168 | unittype_Med-Surg ICU | 169 | unittype_Neuro ICU | 170 | unittype_SICU |
| 171 | unitadmitsource_Acute Care/Floor | 172 | unitadmitsource_Chest Pain Center | 173 | unitadmitsource_Direct Admit |
| 174 | unitadmitsource_Emergency Department | 175 | unitadmitsource_Floor | 176 | unitadmitsource_ICU |
| 177 | unitadmitsource_Operating Room | 178 | unitadmitsource_Other Hospital | 179 | unitadmitsource_Other ICU |
| 180 | unitadmitsource_PACU | 181 | unitadmitsource_Recovery Room | 182 | unitadmitsource_Step-Down Unit (SDU) |
| 183 | unitstaytype_admit | 184 | unitstaytype_readmit | 185 | unitstaytype_transfer |
| 186 | physicianspeciality_Specialty Not Specified | 187 | physicianspeciality_anesthesiology | 188 | physicianspeciality_anesthesiology/CCM |
| 189 | physicianspeciality_cardiology | 190 | physicianspeciality_critical care medicine (CCM) | 191 | physicianspeciality_emergency medicine |
| 192 | physicianspeciality_endocrinology | 193 | physicianspeciality_family practice | 194 | physicianspeciality_gastroenterology |
| 195 | physicianspeciality_hematology | 196 | physicianspeciality_hematology/oncology | 197 | physicianspeciality_hospitalist |
| 198 | physicianspeciality_infectious disease | 199 | physicianspeciality_internal medicine | 200 | physicianspeciality_nephrology |
| 201 | physicianspeciality_neurology | 202 | physicianspeciality_nurse practitioner | 203 | physicianspeciality_obstetrics/gynecology |
| 204 | physicianspeciality_oncology | 205 | physicianspeciality_orthopedics | 206 | physicianspeciality_other |
| 207 | physicianspeciality_otolaryngology | 208 | physicianspeciality_physical medicine/rehab | 209 | physicianspeciality_psychiatry |
| 210 | physicianspeciality_pulmonary | 211 | physicianspeciality_pulmonary/CCM | 212 | physicianspeciality_radiology |
| 213 | physicianspeciality_rheumatology | 214 | physicianspeciality_surgery-cardiac | 215 | physicianspeciality_surgery-critical care |
| 216 | physicianspeciality_surgery-general | 217 | physicianspeciality_surgery-neuro | 218 | physicianspeciality_surgery-orthopedic |
| 219 | physicianspeciality_surgery-plastic | 220 | physicianspeciality_surgery-transplant | 221 | physicianspeciality_surgery-trauma |
| 222 | physicianspeciality_surgery-vascular | 223 | physicianspeciality_unknown | 224 | physicianspeciality_urology |
| 225 | > 89 |  |  |  |  |

1. Visualization of Model Performance Across 5 Days: Feature Activation Trends and Insights"


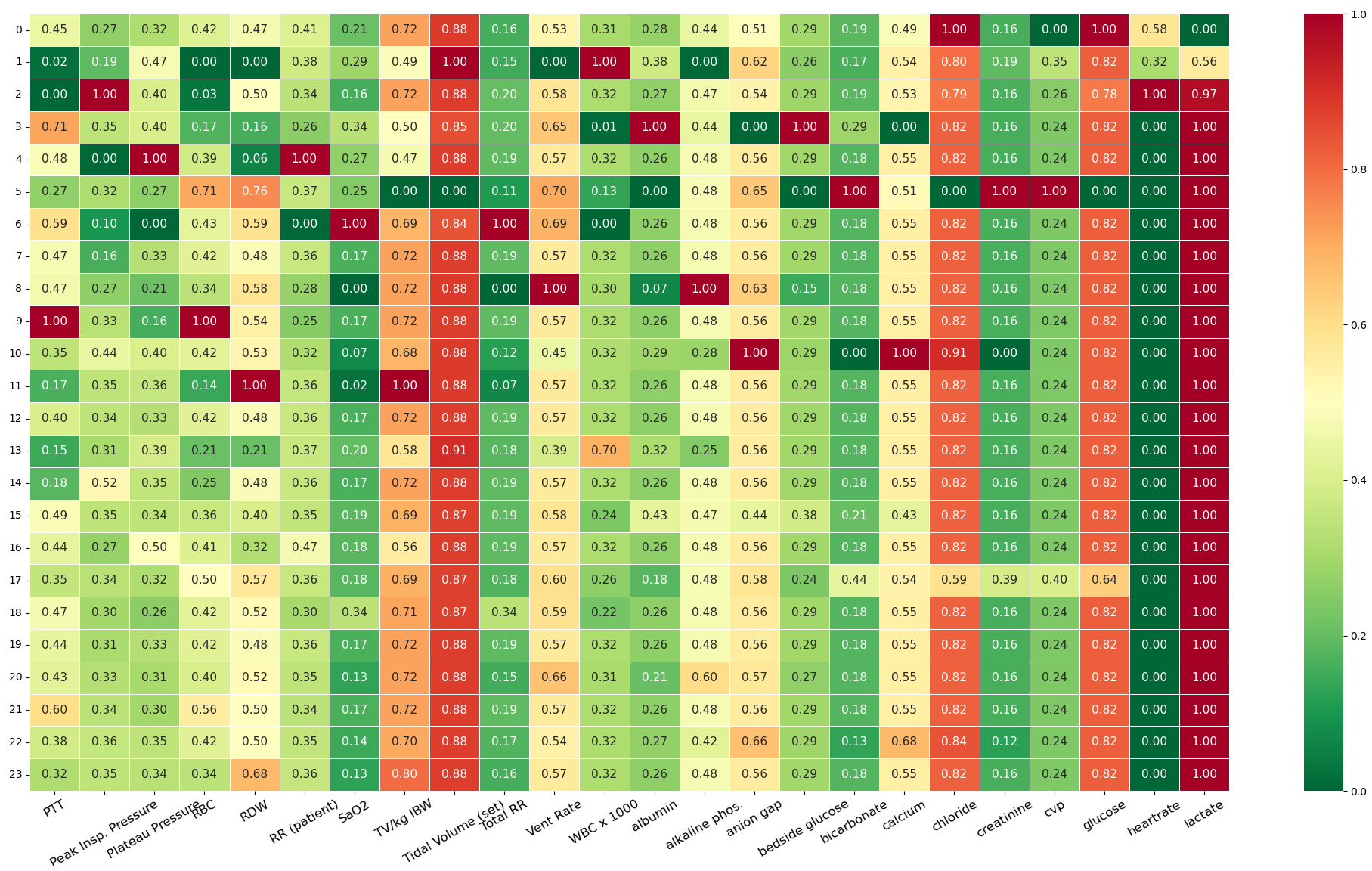


**Figure 1. Day-1 model visualization results, with prominently activated features including** **Lactate, tidal volume, chloride, glucose**


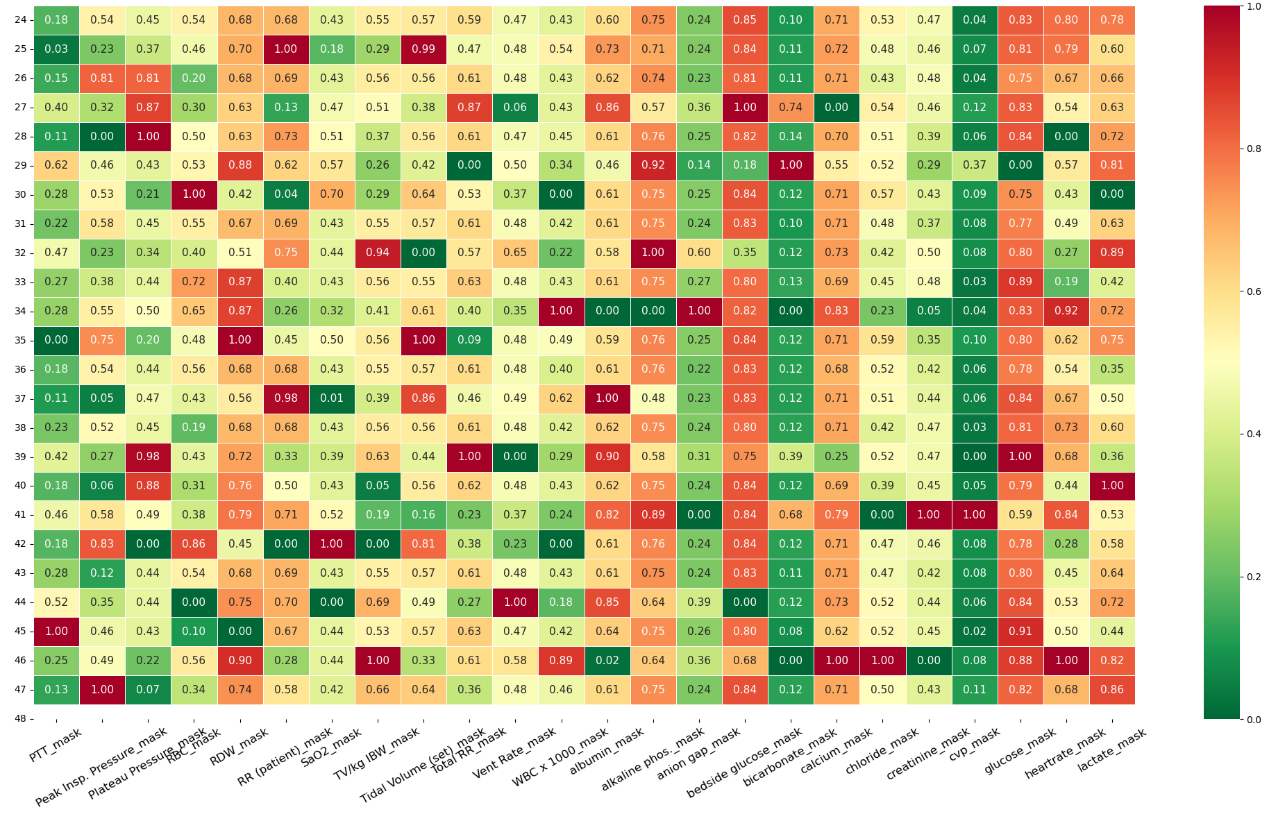
 **Figure 2. Day-2 model visualization results, with prominently activated features including**: **RDW, albumin, alkaline, glucose, calcium**
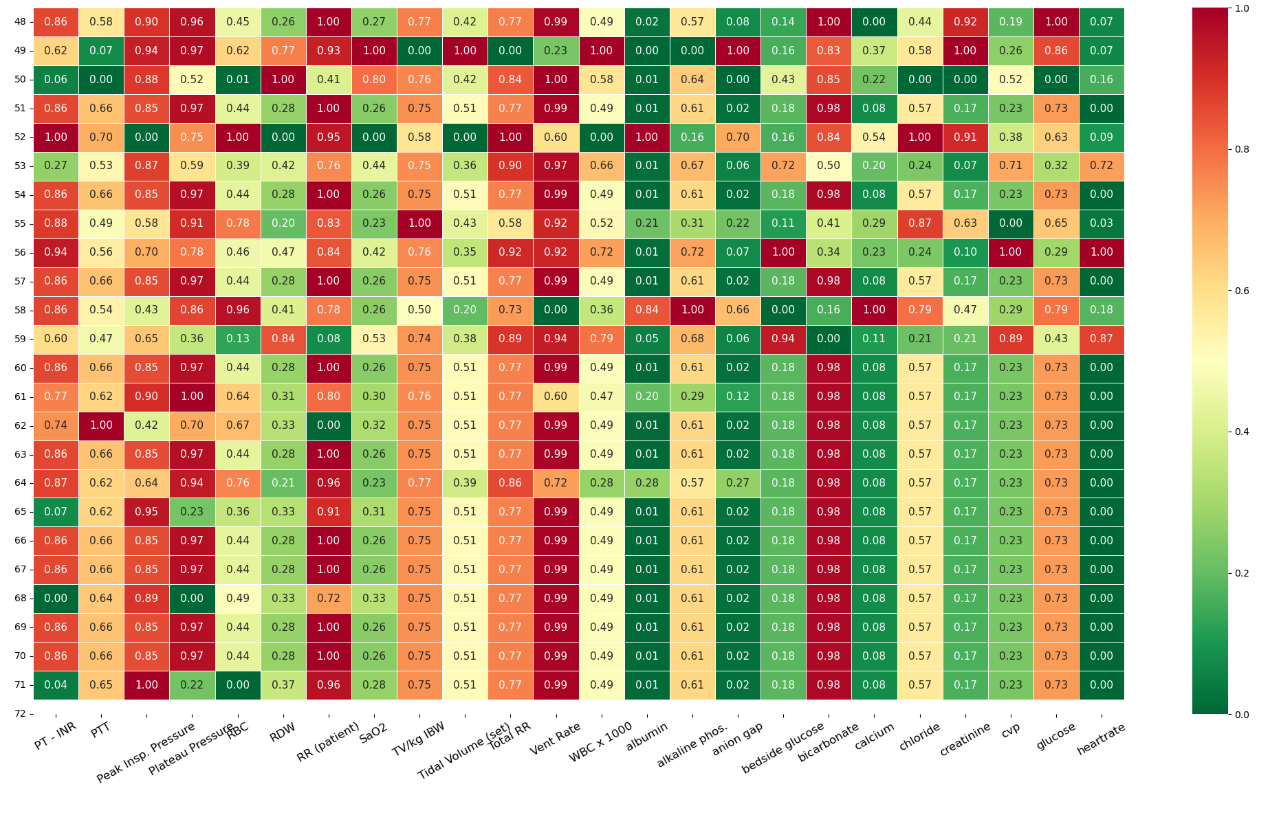


**Figure 3. Day-3 model visualization results, with prominently activated features including**: **PT-INR, PTT, Peak Insp.Pressure, plateau pressure, Total RR, TV/kg IBW, Total RR, Vent Rate, bicarbonate**


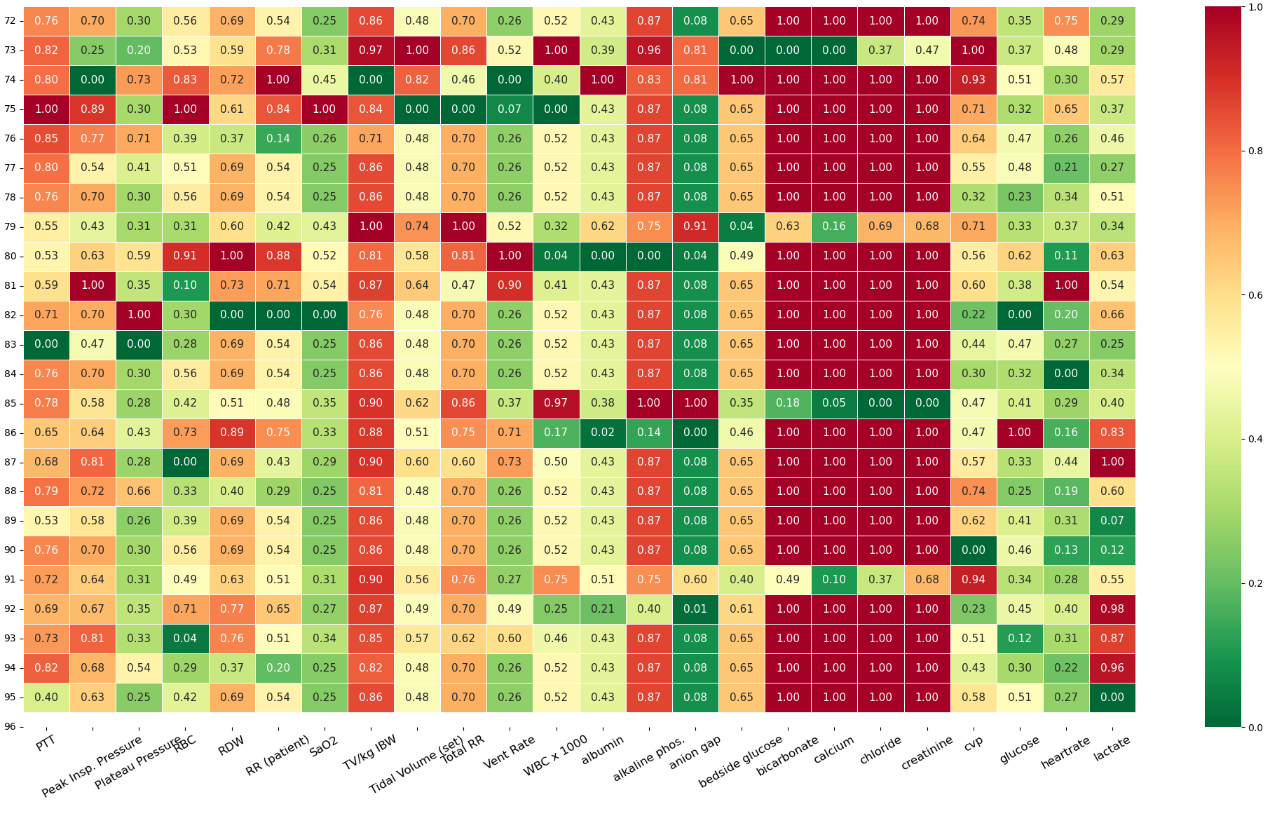


**Figure 4. Day-4 model visualization results, with prominently activated features including**: **PTT, Peak Insp.Pressure, TV/kg IBW, Total RR, creatine, chloride, calcium, bicarbonate**


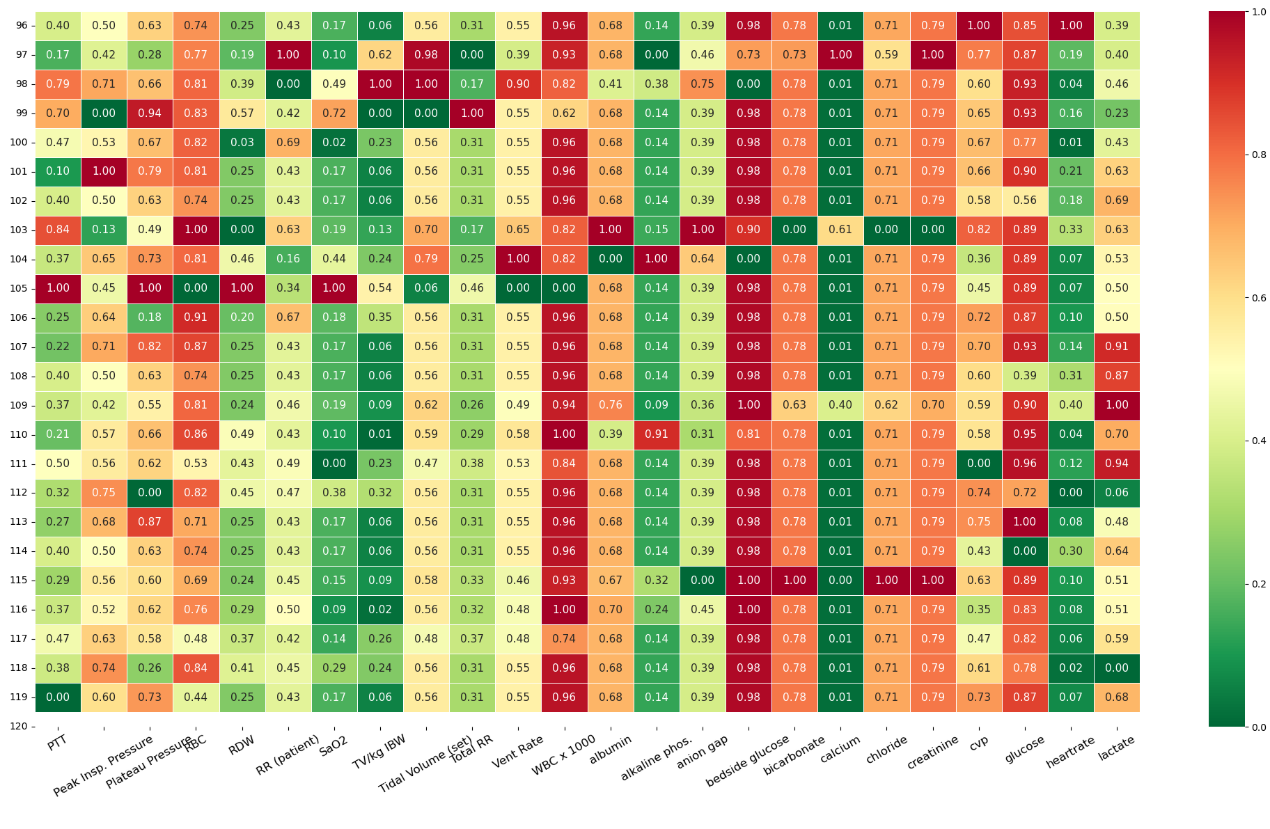
 **Figure 5. Day-5 model visualization results, with prominently activated features including**: **RBC, WBC, glucose, bicarbonate, chloride, creatinine**

1. Model Performance Comparison: Bar Chart of AUC, Accuracy, and F1 Metrics

**Figure 6. Model Performance Comparison: Bar Chart of AUC, Accuracy, and F1 Metrics (*P*<0.001)**

**Figure 7. The outcome of external validation**

**Figure 8. The outcome of external validation in MIMIC**

**Figure 9. The framework of Hospital System Integration with Predictive Models**
